# Emergency surgery score as a predictor of postoperative surgical site infection in patients undergoing emergency surgery for gastrointestinal diseases: A prospective cohort study

**DOI:** 10.12669/pjms.42.6.15726

**Published:** 2026-06

**Authors:** Umama Ehtesham, Yusra Khan, Muhammad Tayyab ul Hassan Siddiqui, Fariha Ashraf, Ghulam Murtaza

**Affiliations:** 1 Dr. Umama Ehtesham (MBBS, FCPS), Department of General Surgery, Patel Hospital, Karachi, Pakistan; 2 Dr. Yusra Khan (MBBS, FCPS), Department of General Surgery, Patel Hospital, Karachi, Pakistan; 3 Dr. Muhammad Tayyab ul Hassan Siddiqui, MBBS, FCPS(GS), FEBS(Surgical Oncology), Consultant Surgical Oncology, Patel Hospital, Karachi, Pakistan; 4 Dr. Fariha Ashraf (MBBS, FCPS), Department of General Surgery, Patel Hospital, Karachi, Pakistan; 5 Dr. Ghulam Murtaza (FCPS, MRCS, MSC, FACS), Department of General Surgery, Patel Hospital, Karachi, Pakistan

**Keywords:** Emergency Surgery, Emergency Surgery Score, Low Middle Income Country, Risk Stratification, Surgical Site Infection

## Abstract

**Objectives:**

To measure the predictability of Emergency Surgery Score (ESS) in development of postoperative surgical site infections (SSIs) in emergency gastrointestinal surgery, during a period of 30 day follow up.

**Methodology:**

In this prospective cohort study conducted at Patel Hospital between December 2023-January 2025, adults undergoing emergency gastrointestinal surgery for perforation, obstruction, inflammation, or ischemia were enrolled. Preoperative ESS was calculated. Patients with early mortality, extra-gastrointestinal infections, or immunocompromised status were excluded. The association between ESS and SSI was analyzed after a 30-day follow-up.

**Results:**

Of fifty-six patients (median age 27.5 years; 69.6% male), the SSI incidence was 26.8% (n=15). Acute appendicitis (55.4%) was the most common diagnosis. SSI incidence rose significantly with ESS: 9.1% (ESS 0), 20% (ESS 1–3), and 70% (ESS 4–29). Logistic regression identified ESS 4–29 (OR: 23.3; p=0.028) and open procedures (OR: 23.1; p<0.001) as significant predictors. ROC analysis showed an Area under the Curve of 0.704 (p=0.008). An ESS cutoff of ≥3 provided 93% specificity and 47% sensitivity. Each 1-point ESS increase raised SSI odds by 1.45 times (95% CI: 1.11–1.90).

**Conclusion:**

The ESS is a straightforward bedside tool that incorporates physiological and disease-related risk factors. An ESS cutoff of three or more showed high specificity, supporting its use for preoperative risk stratification and targeted infection prevention in emergency gastrointestinal surgery, especially in resource-limited settings.

## INTRODUCTION

Emergency surgery refers to surgical intervention undertaken in response to an immediate threat to life, organ function, or tissue viability. The overall burden of emergency surgical disease has become substantial and continues to increase, with rates exceeding those seen for several fatal conditions, including cancer.^1^ A significant proportion of non-communicable disease burden is amenable to surgical treatment, and the cumulative mortality associated with surgically treatable conditions exceeds that of infectious diseases, maternal and peri-natal causes, and malnutrition combined.^2^ A global meta-analysis of over 488,000 patients found a pooled 30 days surgical site infection incidence of 11%, identifying surgical duration and emergency status as key factors influencing infection risk.^3^ In general, emergency surgical procedures are associated with higher morbidity and mortality compared with elective surgery.^4^

This increased risk is attributable to delayed presentation and advanced disease at the time of intervention.^4^ Patients with emergency gastrointestinal conditions often present with complicated pathology such as obstruction, ischemia, perforation, or peritonitis, which is associated with higher postoperative infectious morbidity compared with elective surgery, making surgical site infection (SSI) a key determinant of outcomes.^4^ SSI rates are consistently higher in low- and middle-income countries due to emergency presentation, limited peri-operative optimization, and resource constraints.^5^,^6^

In Pakistan and similar settings, the predominance of infectious etiologies for example typhoid fever, further increases intra-operative contamination and sepsis risk, thereby amplifying postoperative infection rates.^7^ Emergency surgery has been identified as an independent risk factor for postoperative morbidity and mortality, prompting the development of multiple risk stratification tools aimed at predicting outcomes in this high-risk population.^8^ Commonly used systems include the American Society of Anesthesiologists (ASA) classification, the American College of Surgeons National Surgical Quality Improvement Program (ACS-NSQIP), the Physiological and Operative Severity Score for the Enumeration of Mortality and Morbidity (POSSUM), and the Surgical Risk Scale (SRS). However, many of these tools are complex or require postoperative variables, limiting their utility in time-sensitive emergency settings.

The Emergency Surgery Score (ESS) was developed as a preoperative risk assessment tool to predict postoperative complications and mortality in patients undergoing emergency surgery.^9^ Prior validation studies have demonstrated that increasing ESS values are associated with progressively higher rates of postoperative morbidity and mortality, with mortality rising from negligible levels at low scores to substantial rates at higher scores.^9^-^11^ More recent work has shown that ESS retains predictive performance even when one or more data elements are missing, supporting its practicality in real-world emergency setting.^10^-^12^

Although ESS has been validated as a predictor of morbidity, mortality, and postoperative infections in general emergency surgical populations, most existing evidence originates from high-income settings and includes heterogeneous case mixes. Emergency gastrointestinal conditions represent a clinically distinct subgroup with a particularly high burden of surgical site infection, especially in LMICs where delayed presentation and infection-related pathology are common. Prospective data evaluating the performance of ESS specifically for predicting postoperative infectious complications in emergency bowel surgery within LMIC settings remain limited. Our objective was to measure the predictability of Emergency Surgery Score (ESS) in development of postoperative surgical site infections (SSIs) in emergency intestinal surgery, during a period of 30-day follow-up, in order to validate it as a preoperative metric for identifying high-risk patients for developing SSI.

## METHODOLOGY

This prospective cohort study was conducted in the Department of General Surgery at Patel Hospital, Karachi, a tertiary-care teaching hospital, from January 2024 to January 2025. Adult patients presenting to the emergency department with small or large bowel pathologies, including perforation, ischemia, or obstruction, requiring emergency surgical intervention, were eligible for inclusion. Patients who died within 36 hours of presentation, had extra-intestinal infection at admission, were immunocompromised (including those receiving chemotherapy or radiotherapy), or were on immunosuppressive medications were excluded. Participants were enrolled using consecutive sampling. The primary outcome was postoperative surgical site infection (SSI), defined and classified as superficial incisional, deep incisional, or organ-space infection according to Centers for Disease Control and Prevention criteria. The main exposure variable was the Emergency Surgery Score (ESS), calculated preoperatively using clinical and laboratory parameters. Demographic characteristics, co morbidities, operative findings, and microbiological culture results were recorded using a standardized data collection proforma. Sample size was calculated using WHO statistical software, assuming an expected SSI rate of 3.8%,^13^ with a 95% confidence interval and a 5% margin of error, yielding a required sample size of 56 patients. To reduce selection bias, all eligible patients presenting during the study period were consecutively enrolled.

### Ethical Approval:

Approval was obtained from the institutional Ethical Review Committee (Ref: PH/IRB/2023/024; dated September 15, 2023).

### Patient consent:

Consent of the patient/guardian was taken prior to ESS calculation and in context of the study.

### Emergency Surgery Score (ESS):

The Emergency Surgery Score is a validated preoperative risk assessment tool comprising 22 clinical, demographic, and laboratory variables, with a maximum cumulative score of 29 points (Appendix-I). The ESS was calculated pre-operatively by a general surgery resident based on information obtained from the patient, accompanying family member, and hospital records.

**APPENDIX-1:** Emergency Surgery Score.

Variable	Points
**Demographics**	
Age >60 y	2
White race	1
Transfer from outside emergency department	1
Transfer from an acute care hospital inpatient facility	1
**Co-morbidities**	
Ascites	1
BMI <20 kg/m2	1
Disseminated cancer	3
Dyspnea	1
Functional dependence	1
History of COPD	1
Hypertension	1
Steroid use	1
Ventilator requirement within 48 h pre-operatively	3
Weight loss > 10% in the preceding 6 mo	1
**Laboratory values**	
Albumin <3.0 U/L	1
Alkaline phosphatase >125 U/L	1
Blood urea nitrogen >40 mg/dl	1
Creatinine >12 mg/dl	2
International normalized ratio >15	1
Platelets <150×103 mcL	1
SGOT >40 U/L	1
Sodium >145 mg/dl	1
WBC X 103 mcL	
<4.5	1
>15 and ≤25	1
>25	2
Maximum score	29

BMI body mass index; COPO=chronic obstructive pulmonary disease; SGOT-serum glutamic oxaloacetic transaminase: WBC = white blood cell.

### Data collection:

After obtaining informed written consent, a structured performa was used to collect data. Patients were first evaluated in the emergency room, where clinical history, comorbidities, and initial laboratory work were documented. The preoperative section of the proforma was completed in the operating room’s preoperative area. Postoperative follow-up was conducted by general surgery residents either in the intensive care unit or surgical wards. Daily wound evaluations were carried out until discharge. Upon discharge, patients received detailed instructions regarding wound care, which included either removal of dressings, in case of laparoscopic procedures, or daily midline wound dressings and evaluation for signs of SSI. They were advised scheduled follow-up visits for a 30-day postoperative period, that included one regular follow up visit required post operatively after one week, and the rest of the follow ups were dictated by the clinical condition of the patient and the wound. In cases where patients were discharged before full recovery, their contact details were recorded to facilitate outpatient follow-up. Patient confidentiality was maintained throughout the study duration.

### Data analysis:

Data were analyzed using SPSS, Version 27.0. Continuous variables were assessed for normality; non-normally distributed data (e.g., Age, ESS) were presented as medians with inter quartile ranges (IQR). Categorical variables (e.g., ESS Categories, Gender, Diagnosis, Presence of Co morbidity) were expressed as frequencies and percentages (n, %).

The association between the ESS and the development of SSI was evaluated using the Chi-square test for categorical variables. This analysis specifically compared the stratification of ESS scores between patients with and without SSI. Binary logistic regression analysis was performed to identify independent predictors of SSI. It included variables such as ESS categories, Diabetes, procedure type (Laparoscopic vs. Open), diagnosis, and gender. Results were reported as Odds Ratios (OR) with 95% Confidence Intervals (CI) and p-values. A p-value of <0.05 was considered statistically significant.

Receiver Operating Characteristic (ROC) curve analysis was conducted to evaluate the discriminatory power of the Emergency Surgery Score (ESS) in predicting SSI. The Area under the Curve (AUC) was calculated. The optimal cutoff point for the ESS was determined value using Youden’s Index, to maximize specificity and sensitivity, and diagnostic accuracy metrics (Sensitivity, Specificity) were reported.

## RESULTS

A total of 56 patients were included in the study. The median age of the cohort was 27.5 years (IQR: 19–47). The majority of participants were male (69.6%). The most common diagnosis was acute appendicitis (55.4%), followed by perforated appendicitis (19.6%). Most patients (69.9%) had no comorbidity, while diabetes (10.7%) and hypertension (7.1%) were the most common medical conditions The overall incidence of postoperative Surgical Site Infection (SSI) was 26.8% (n=15). More than half of the cultures showed no growth (55.4%), whereas *E. coli* was the most common organism in culture-positive samples (Table-I).

**Table-I:** Baseline demographics and clinical characteristics of patients undergoing emergency intestinal surgery (N=56)

Variable	Value
Age, years, median (IQR)	27.5 (19-47)
***Gender, n (%)***	
Male	39 (69.6)
Female	17 (30.4)
***Presence of comorbidity, n (%)***	
None	39 (69.6)
Diabetes mellitus	6 (10.7)
Diabetes + hypertension	4 (7.1)
Other	7 (12.3)
Emergency Surgery Score (ESS), median (IQR)	1 (1–3)
ESS range	0–29
***Diagnosis, n (%)***	
Acute appendicitis	31 (55.4)
Perforated appendicitis	11 (19.6)
Peptic-ulcer related perforations	6 (10.7)
Small bowel obstruction	4 (7.1)
Sigmoid perforation	2 (3.6)
Anastomotic leaks	2 (3.6)
Typhoid perforation	1 (1.8)
Gastric outlet obstruction	1 (1.8)
Appendicitis with worm infestation	1 (1.8)
***Cultures, n (%)***	
No growth	31 (55.4)
Culture-positive	25 (44.6)
Escherichia coli (E. coli) only	7 (12.5)
E. coli + Candida albicans	2 (3.6)
Klebsiella + MRSA + S. pneumonia	1 (1.8)
Enterococcus VRE	1 (1.8)
Other positive cultures*	14 (25.0)
***SSI n (%)***	
Developed SSI	15 (26.8)
No SSI	41 (73.2)

**Fig.1:**
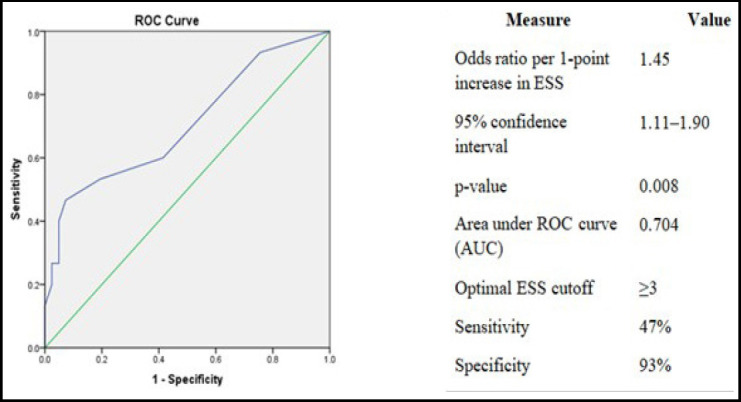
Receiver Operating Characteristic (ROC) Curve Analysis and Diagnostic Performance of ESS in Predicting SSI.

Table-II demonstrates the distribution of ESS scores stratified by SSI outcome. There was a clear trend showing higher infection rates with higher ESS scores. The ROC curve analysis (Fig.1) assessed the ability of the ESS to predict SSI. The ESS demonstrated fair predictive ability with an Area under the Curve (AUC) of 0.704 (*p* = 0.008). An ESS of ≥3 was identified as the optimal cutoff. At this cutoff, the score provided a Specificity of 93% and a Sensitivity of 47%. The odds of SSI increased by a factor of 1.45 for every 1-point increase in the ESS. On the basis of which ESS was categorized as ESS 0: Only 9.1% of patients developed SSI. ESS 1-3: 20% of patients developed SSI. ESS 4-29: The infection rate rose dramatically to 70% (7 out of 10 patients in this category).

**Table-II:** Cross-tabulation of Emergency Surgery Score (ESS) Categories and Incidence of Surgical Site Infection (SSI).

		SSI	
		No	Yes
ESS n (%)	0	10 (90.9)	1 (9.1)
	1	14 (73.7)	5 (26.3)
	2	9 (90)	1 (10)
	3	5 (83.3)	1 (16.7)
	4	1 (50)	1 (50)
	5	0 (0)	2 (100)
	6	1 (100)	0 (0)
	7	0 (0)	1 (100)
	8	1 (50)	1 (50)
	10	0 (0)	1 (100)
	13	0 (0)	1 (100)
ESS Categories n (%)	0	10 (90.9)	1 (9.1)
	1-3	28 (80)	7 (20)
	4-29	3 (30)	7 (70)

In the logistic regression analysis (Table-III), several factors were evaluated for their association with SSI. Categorically, Patients with an ESS between 4–29 (OR: 23.3; 95% CI: 1.9–273.2; *p* = 0.028) had significantly higher odds of developing SSI compared to those with a score of 0, and score 1-3 (OR: 2.5; 95% CI: 0.2-22.9; *p* =0.418). There was a positive trend of developing SSI with a score of 1-3, yet it was not found to be statistically significant. Open procedures were strongly associated with SSI compared to laparoscopic procedures (OR: 23.1; 95% CI: 4.3–121; *p*< 0.001). The diagnosis of viscus perforation has a significant association with an Odds Ratio (OR) of 18.7 (95% CI: 3.4-103; p < 0.001) for developing SSI. Diabetes showed a trend toward increased risk (OR: 4.6) but did not reach statistical significance (p = 0.068).

**Table-III:** Logistic regression analysis of risk factors associated with postoperative surgical site infection.

Variable	OR	CI	p-Value
***ESS categories***			
0	-	-	-
1-3	2.5	0.2-22.9	0.418
4-29	23.3	1.9-273.2	0.028
***Co morbidity***			
Diabetes	4.6	0.89-23.7	0.068
***Procedure***			
Laparoscopic	-	-	-
Open Procedure	23.1	4.3-121	<0.001
***Diagnosis***			
Inflammation	-	-	-
Perforation	18.7	3.4-103	<0.001
Obstruction	15.0	1.7-128	0.013
***Gender***			
Female	-	-	-
Male	1.27	0.34-4.77	0.717

## DISCUSSION

In this prospective cohort of patients undergoing emergency surgery for gastrointestinal diseases, elevated Emergency Surgery Score (ESS) values were significantly associated with an increased risk of postoperative surgical site infection (SSI). Most notably, we observed a stepwise progression in risk: for every single-point increase in the ESS, the odds of developing an SSI rose by approximately 45%. The score demonstrated acceptable discriminatory capability (AUC 0.704), with a cutoff of ≥3 providing high specificity. This threshold effectively creates a subset of patients at significantly high risk of developing SSI, validating the ESS as a practical tool for SSI prediction in this population, and subsequently caters to devise a suitable management plan for patients.

While our results align with broader international trends, this study adds to the growing body of evidence in validating ESS in the South Asian region and in our specific local institutional context. Our observation of a 45% risk increase in SSI per ESS point provides a more granular metric than the broader categories suggested by Nandan et al.^9^ where ESS was validated as a predictor of overall postoperative complications. Our results, also, align with Han et al.,^13^ who reported a similar stepwise increase in infectious complications with rising ESS. Although previous studies did not always report SSI-specific ROC metrics, they collectively establish ESS as a clinically authentic predictor of infectious outcomes in emergency general surgery.

The strength of the ESS is supported by data from diverse healthcare settings, particularly in low- and middle-income countries (LMIC). Similarly, Montasser et al. and simultaneous studies^14^-^16^ have confirmed the utility of ESS for predicting adverse outcomes in emergency laparotomy cohorts. Although not all focused exclusively on SSI, these studies support the applicability of ESS as a risk stratification tool in resource-limited settings.

Validation studies across the USA, UK,^15^ Greece,^17^ Jordan^18^ and Saudi Arabia^19^ further cement the ESS as a reliable global metric. Recent analyses by Ghali et al. and others^17^,^20^,^21^ found that ESS reliably predicted postoperative complications, ICU admission, and mortality across diverse populations, including the Middle East and North Africa (MENA) cohorts. These findings complement our focus on SSI and support the continued investigation of ESS in settings where simple bedside risk assessment is critical.^16^

Risk stratification is particularly important in LMICs, which hold a disproportionately high burden of SSI. The GlobalSurg Collaborative demonstrated a clear gradient in SSI rates, with the highest incidence in low development index countries.^22^ In Pakistan, Afridi et al.^7^ noted that perforation peritonitis constitutes a major volume of emergency abdominal surgery and drives substantial infectious morbidity, while Khan et al.^5^ identified contaminated emergency procedures as major predictors of SSI. By integrating physiological derangement with disease severity, the ESS provides an objective gradient of risk, distinguishing low-risk patients from those facing high mortality and morbidity, consistent with large-scale multi-institutional findings.^19^-^23^

In the present cohort, severe intra-abdominal pathologies specifically hollow viscus perforation correlated with higher SSI rates, contrasting with the minimal morbidity observed in uncomplicated appendicitis. This reinforces that while the ESS does not replace anatomical diagnosis, it effectively integrates physiological derangement with disease severity to identify high-risk phenotypes.

### Strength of the study:

This study’s practical attention to detail is a primary strength, characterized by a prospective design, complete 30-day follow-up, and standardized outcome assessment using CDC criteria. The identification of a specific ESS cutoff (≥3) enhances its clinical utility, transforming the score into an actionable bedside tool. By testing the ESS in this specific environment, we’ve shown that a lack of resources doesn’t have to mean a lack of precision. This research helps bridge a long-standing divide in global surgery, providing a feasible way to identify patients who are more prone to develop SSI, when every clinical decision counts.

### Limitations:

Despite the clinical relevance of these results, we encountered a few practical limitations during the study. The expected rate of SSI being 3.8%, from the primary article^13^ was not adequately powered and hence our infection rate was therefore 26.8%. While our findings are vital, the study has a small sample size, and hence did present some statistical hurdles. In certain diagnostic groups, the outcomes were so consistent that our multivariable models couldn’t reach a stable conclusion—a technical issue known as ‘complete separation’ that often occurs in smaller cohorts. Additionally, age was excluded from the final regression to avoid collinearity, as it is an inherent component of the ESS itself. Despite these statistical constraints, the strong univariable association confirms the ESS’s predictive validity, aiding surgical fraternity to predict their patient outcomes by way of devising patient specific management plans. Ultimately, this study establishes the ESS as a valuable adjunct for preoperative risk assessment in emergency intestinal surgery, warranting further validation through larger, multicenter trials to refine risk thresholds.

## CONCLUSION

ESS provides a simple bedside tool that integrates physiological and disease-related risk factors in patients undergoing emergency gastrointestinal surgery. An ESS cutoff of ≥3 showed high specificity and accurately predicted 30-day postoperative surgical site infections (SSIs) in emergency gastrointestinal surgery, suggesting clinical utility for preoperative risk stratification. While it should not be viewed as a substitute for clinical judgment or underlying diagnosis, these findings underscore its potential to guide targeted infection prevention strategies, particularly in low- and middle-income settings.

### Authors’ Contributions:

**UE:** Substantial contributions to conception, design, acquisition, analysis and interpretation of data and drafting the article.

**YJ:** Analysis and interpretation of data.

**TUH:** Conception, design, and final approval, Agreement to be accountable for all aspects of the work.

**FA:** Formation of scientific question, Critical review for important intellectual content.

All authors have read and approved the final version. They are also responsible for the integrity and accuracy of the study.
